# Clinical and radiological 2-year results after autologous shaver-based minced cartilage implantation for cartilage lesions of the knee

**DOI:** 10.1007/s00402-025-06010-8

**Published:** 2025-10-10

**Authors:** Stefan Pohl, Matthias Mühler, Alexander Zimmerer, Janosch Schoon, Georgi I. Wassilew, Sebastian Gebhardt

**Affiliations:** 1 Center for Orthopaedics, Trauma Surgery and Rehabilitation Medicine, University Medicine Greifswald, Ferdinand-SauerbruchStraße, 17475 Greifswald, Germany; 2 Institute for Diagnostic Radiology and Neuroradiology, University Medicine Greifswald, Greifswald, Germany; 3 Department of Orthopaedic and Trauma Surgery, Orthopädische Klinik Paulinenhilfe, Diakonieklinikum Stuttgart, Stuttgart, Germany

## Abstract

**Introduction:**

The autologous shaver-based minced cartilage implantation is increasingly used for the treatment of focal cartilage lesions of the knee. However, in the absence of randomised controlled studies on this specific technique, its efficacy in comparison to established cartilage repair methods is a matter of debate.

**Materials and methods:**

Eleven patients (12 cases) were prospectively included in this study. One female patient was excluded due to conversion to total knee arthroplasty. All 11 analysed cases (8 females, 3 males; mean age: 42.7 ± 11.8 years, body mass index (BMI): 29.6 ± 6.1 kg/m^2^, lesion size: 3.2 ± 1.6 cm^2^) received autologous shaver-based minced cartilage implantation (AutoCart™, Arthrex Inc.) due to full thickness cartilage lesion of the knee. The patients were evaluated preoperatively and at 6, 12- and 24-months follow-up, using the International Knee Documentation Committee (IKDC) Questionnaire, the Knee Injury and Osteoarthritis Outcome Score (KOOS) and Visual Analog Scale (VAS) pain. The minimal clinically important difference (MCID) was calculated using the half-standard deviation method. Magnetic resonance imaging (MRI) was performed after 24 months and evaluated using the Magnetic resonance Observation of Cartilage Repair Tissue grading scale (MOCART) and the calculation of a T2-Index comparing T2 relaxation times of transplanted cartilage with intact cartilage of the same joint.

**Results:**

Statistically significant improvement at 24 months follow up was observed for pain relief, as documented by the VAS and KOOS total, as well as the KOOS subsccores pain and activities of daily living. Out of 11 cases the MCID was reached in 9 cases considering VAS, 5 cases considering IKDC, 7 cases considering KOOS total and sport, 6 cases considering quality of life and 5 cases considering symptoms and activities of daily living. The postoperative mean MOCART score was 51.4 ± 22.0 and the mean T2-Index was 0.72 ± 0.18. In 3 of 11 postoperative MRIs no transplanted cartilage was observed.

**Conclusion:**

Treatment of focal cartilage lesions at the knee with the autologous shaver-based minced cartilage implantation provided pain relief and improved function at 2 years follow up. Radiologic results indicated that on average the transplanted cartilage was different from hyaline cartilage, however, according to the T2-Index, in two cases the transplanted cartilage exhibited qualitative MR morphological properties similar to hyaline cartilage.

**Supplementary Information:**

The online version contains supplementary material available at 10.1007/s00402-025-06010-8.

## Introduction

As a promising alternative treatment method for focal cartilage lesion of the knee, the autologous shaver-based minced cartilage implantation has recently attracted attention. The first generation of minced cartilage implantation (MCI) has been described as an open surgery at the knee joint in 1983 [1]. The currently evolved autologous shaver-based minced cartilage implantation is a continued development of MCI and allows for a minimal invasive, arthroscopic and completely autologous procedure, by utilizing an arthroscopic shaver for harvesting and mincing of the cartilage tissue, patient specific autologous conditioned plasma (ACP) for augmentation of cartilage tissue and autologous thrombin to fixate the implanted cartilage tissue into the lesion. The autologous shaver-based minced cartilage implantation therefore has distinct advantages compared to established cartilage repair techniques. In contrast to microfracturing (MFX) or autologous matrix induced chondrogenesis (AMIC), the autologous shaver-based minced cartilage implantation transplants autologous cartilage tissue. Frequently reported disadvantages of MFX like intralesional osteophyte formation or fibrocartilage formation might not account for the autologous shaver-based minced cartilage implantation [2]. Compared to the autologous chondrocyte transplantation (ACT), that today is the only other available cartilage tissue transplanting technique, autologous shaver-based minced cartilage implantation has the advantages of being a one step procedure and has lower treatment costs [3]. However, for the ACT superiority over MFX in randomized controlled trials [4–6] as well as long term efficacy is documented [7]. For the autologous shaver-based minced cartilage implantation on the other hand, evidence is scarce. Recently published studies on the autologous shaver-based minced cartilage implantation reported favorable clinical and radiological outcomes [8–10]. The present study complements these clinical findings. Furthermore, for the first time, all post-operative MRI protocols included T2 mapping sequences, to gain insights into the qualitative composition of the regenerated cartilage tissue. We hypotesized that treatment of focal cartilage lesions of the knee will lead to a significant reduction of pain and improvement of knee function without adverse events and MRI investigation will indicate transplanted cartilage tissue being similar to hyaline cartilage at 24 months follow up.

## Materials and methods

### Patient recruitment

The study protocol was approved by the local ethics committee of University Medicine Greifswald (Internal Reg. No. BB05/20 (for clinical investigation) and BB030/21 (for radiological investigation) and conducted in accordance with the Declaration of Helsinki. All patients signed informed consent prior to participation in the study. Between September 2020 and July 2021 eleven patients (12 knees) with symptomatic, focal cartilage lesions of the knee joint were included in the present study. All patients were consecutively recruited through two trained physicians (C.J. and S.G.) in one orthopaedic centre. Prerequisite for participation was a patient age of 18 years or older, focal full thickness cartilage lesions of the knee joint (ICRS grade ≥ III) of at least 1cm^2^ in size and written consent to participate in the study. Exclusion criteria were osteoarthritis (> grade II according to Kellgren and Lawrence), previous cartilage surgery or infection of the knee joint, as well as rheumatoid arthritis. Patients were screened for leg axis deviations < 3° tolerated), ligament injuries, meniscus injuries, patella malalignment (Tibial Tuberosity—Trochlea Groove Distance < 20 mm, Tibial Tuberosity—Posterior Cruciate Ligament Distance < 24 mm and Canton Dechamps Index < 1.3 tolerated) and were excluded from the study if concomitant surgery to the autologous shaver-based minced cartilage implantation was indicated. Patients requiring medial patellofemoral ligament reconstruction following first time dislocation of the patella were included.

### Operative technique

Patients were posed in supine position with the leg to be operated on in a leg holder. Preparation included standard sterile washing of the whole leg and sterile draping. All operations were performed in bloodlessness. Initially, standard medial and lateral arthroscopic portals and diagnostic arthroscopy of the joint were performed. The cartilage lesion to be treated was then exposed and debrided using an arthroscopic shaver (3 mm, Sabre, Arthrex Inc.) until a solid rim was created. The AutoCart™ procedure was performed as previously described [11]. In brief, debrided cartilage tissue from the border of the defect was minced by arthroscopic shaver and collected with a designated collection container (GraftNet™, Arthrex Inc.). Autologous conditioned plasma (ACP) was produced simultaneously with the operation from patient blood that was drawn from the antecubital region, using three ACP double syringe systems (Arthrex Inc.). Full blood samples were centrifuged (Horizon 24-AH, Drucker Diagnostics) to generate a supernatant of platelet-rich and leucocyte-poor plasma in the ACP double syringes. Plasma was separated from the cellular portion of the sample within the closed system. The gained ACP was mixed 1:3 (v/v) with the minced cartilage tissue and prepared for reimplantation. Depending on the location of the lesion, the reimplantation of the minced and ACP augmented cartilage tissue was carried out either through the existing arthroscopy portals with the help of a cannula or as part of a mini-open arthrotomy of the joint. During the reimplantation, a defect filling of 80–90% was aspired, as recommended for the surgical procedure described elsewhere [11]. Simultaneously with this procedure, the Thrombinator™ System (Arthrex Inc.) was inoculated with part of the previously obtained ACP and autologous thrombin was produced in this way. The produced autologous thrombin was then instilled onto the transplanted cartilage. Finally, thrombin was mixed with ACP in a 1:1 ratio and this mixture was instilled onto the transplanted cartilage for sealing. A drying time of two minutes was allowed before range of motion control was performed to exclude premature dislocation of the transplanted tissue. The insertion of an intra-articular drain was waived and standard wound closure was performed.

### Postoperative care

Postoperatively the operated leg was wrapped and the operated patients were given 24 h bed rest with the operated knee in full extension. This was followed by mobilization with partial weight-bearing and limited range of motion, which was ensured by the application of a movement-limiting orthosis of the knee joint for 6 weeks postoperatively. Weightbearing was limited to sole contact for the first postoperative week and to 15 kg for the following 5 weeks with progression to full weight bearing thereafter. Range of motion was limited to 30° of flexion for the first and second, 60° of flexion for the third and fourth and 90° of flexion for the fifth and sixth week with progression to full range of movement thereafter. All patients received passive range of motion training with a motorized splint within the above-named motion limitations for six weeks postoperatively. The procedure was carried out uniformly for all patients, regardless of the defect location.

### Patient reported outcome measures

Patient reported outcome measures were obtained prospectively prior to the operation and 6, 12 and 24 months post-operatively. The Visual Analogue Pain Scale (VAS), International Knee Documentation Committee (IKDC questionnaire) [12] and the Knee Injury and Osteoarthritis Outcome Score (KOOS) [13] were obtained. Additionally, a total KOOS Score was calculated by adding the mean of all 5 sub scores (Pain, Symptoms, Activities of Daily Living, Sport and recreation function, Knee-related quality of life) divided by 5. The web based Remote Data Entry (RDE) System (RDE-Light) of the German Arthroscopy Registry (DART) was used for data sampling. With the described system data can be collected directly by an internet browser. The system is based on HTML- and PDF-format. RDE-light is available in various languages, validated according to the good automated practice supplier guide (GAMP^®^5) and fulfils all requirements of good clinical practice. Furthermore, cryptographic security protocols (SSL/TLS), user authentication protocols and authorization concepts are applied as security standard.

### Clinical follow up

A clinical examination was carried out 3 months, 6 months, 12 months and 24 months after the operation using a gyrometric sensor system (Orthelligent^®^, OPED). The system allows for a precise examination of range of motion, stability and proprioception and offers the possibility to draw a comparison to the not operated contralateral leg. The range of motion investigation consisted of passive flexion and extension deficit measurements. Furthermore, dynamic stability and motor control was assessed by the Y-Balance-Test. Prior to testing leg length was measured and subsequently the patient’s ability to reach in anterior, postero-lateral and postero-medial direction with the contralateral leg, while standing on the operated leg was assessed. The final scoring was calculated through the Composite-Score ((anterior reach (cm) + postero-lateral reach (cm) + postero-medial reach(cm)/(leg length (cm) × 3)) × 100).

### MRI follow up

Postoperative MRI of 11 cases was performed 24 months postoperatively. MRI was performed on a 3T whole body scanner (Magnetom VIDA, Siemens Healthineers, Germany) with syngo software (version MR XA50) with a dedicated knee coil (Tx/Rx Knee 18 Tim coil, Siemens Healthineers). For T2 mapping a multi-echo spin echo sequence with 25 slices was applied (TR 1910 milliseconds (ms); TE1 13.8 ms, TE2 27.6 ms, TE3 41.4ms, TE4 55.2 ms, TE5 69 ms; averages 1, concatenations 1, no acceleration, no phase oversampling, voxel size 0.4 × 0.4 × 3.0 mm^2^, acquisition time 6:35 min (min)). Two planes were acquired depending on the localisation of the lesion within the knee joint.

**Fig. 1:**
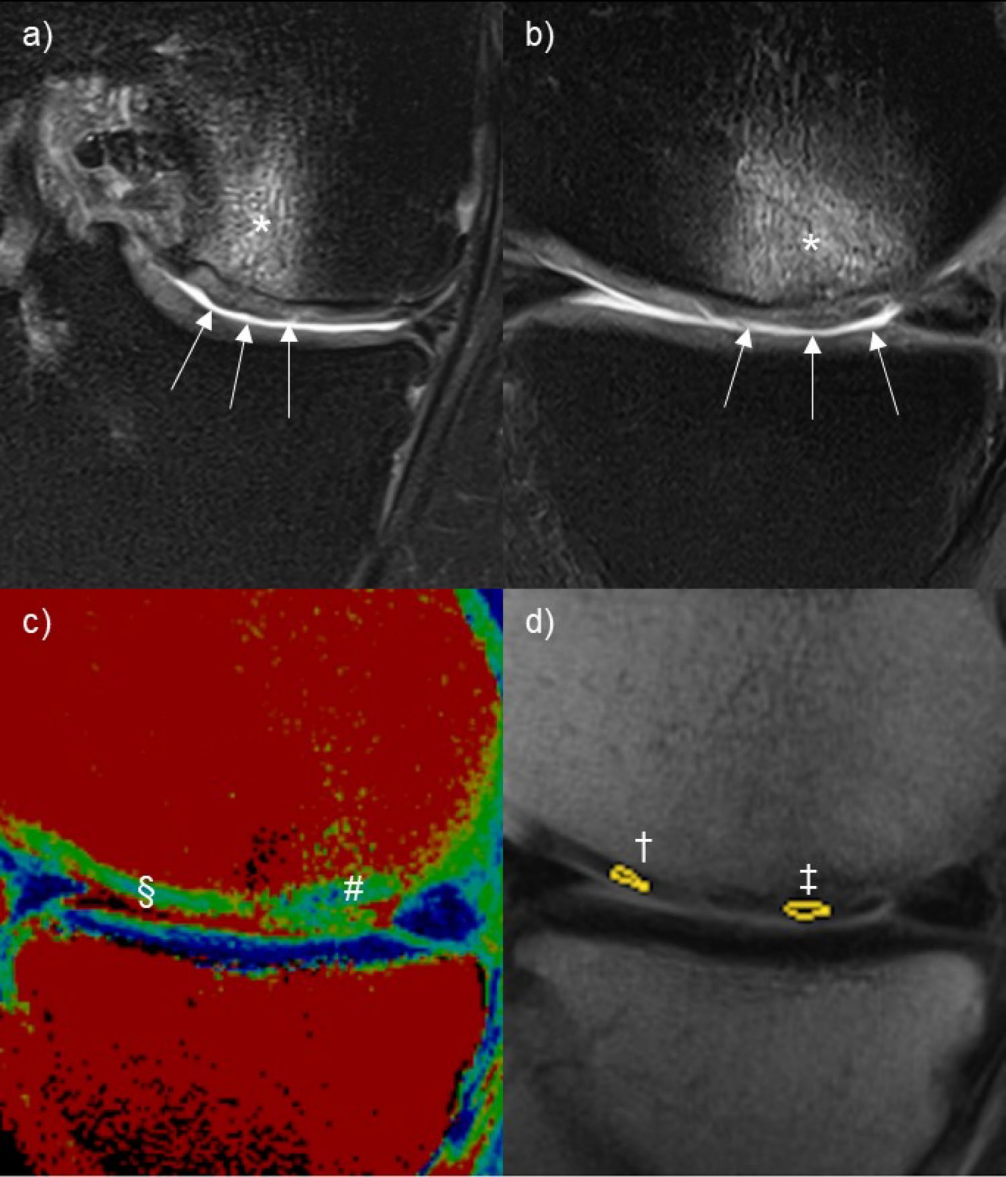
Postoperative MRI of case 3. **a** Coronal Proton Density Turbo Spin Echo sequences (PD TSE) showing the transplanted cartilage (arrows) and associated bone marrow oedema (*). **b** Sagittal PD TSE sequences showing the transplanted cartilage (arrows) and associated bone marrow oedema (*). **c** Sagittal T2 Mapping sequence showing colour coding of transplanted cartilage (^#^) and adjacent healthy hyaline cartilage (^§^). **d** Sagittal T2 Mapping sequence without colour code of transplanted cartilage (^‡^) and adjacent healthy hyaline cartilage (^†^), yellow circles mark the respective region of interest (ROI) in which the relevant T2 relaxation time of included cartilage tissue was measured to calculate the T2-Index

Besides the T2 mapping (Fig. 1c) a standard proton density (PD) weighted turbo spin echo (TSE) sequence with spectral fat saturation (FS) in all three planes (TR 3400 ms, TE 33 ms, magnetization recovery, averages 1, concatenations 2, Grappa factor 3, voxel size 0.2 × 0.2 × 2 mm^3^, 25–35 slices, acquisition time 1:25–2:48 min, Fig. 1a and b) and a standard T1 weighted spin echo (SE) sequence in coronal plane (TR 600 ms, TE 10 ms, averages 2, concatenations 1, SMS acceleration, voxel size 0.3 × 0.3 × 3 mm^2^, 25 slices acquisition time 1:26 min) were applied. The PD FS sequences were acquired with deep learning based Deep Resolve algorithm (Denoising-Modus Boost, Denoising strength medium, Deep Resolve Sharp switched on).

The images were evaluated in consensus reading by a radiologist (M.M.) and an orthopaedic surgeon (S.G.). MRI was read using DeepUnity PACS (version Diagnost 1.1.0.1, Deadalus S.p.A. Milano, Italy). For morphologic evaluation of the cartilage regeneration the Magnetic Resonance Observation of Cartilage Repair Tissue (MOCART) Score was determined as previously described [14]. Furthermore, for the evaluation of T2 mapping, Regions of Interest (ROI) were drawn in areas of regenerated- and intact cartilage of the same joint. The T2-Index was calculated by dividing the T2 relaxation time measured in an ROI within the transplanted cartilage by the T2 relaxation time measured in a ROI within intact cartilage of the same joint (Fig. 1d).

### Statistical analysis

Statistical analysis was conducted with GraphPad Prism Version 9.5.0 (GraphPad Software). Data were assessed for normality by Shapiro–Wilk-Test and are presented as mean and standard deviation or median with Inter Quartile Range (IQR). Appropriate statistical tests were conducted depending on type of distribution: One-way Analysis of Variance (ANOVA) to compare means ± standard deviation (SD) of normally distributed data and multiple comparison Friedman-Test to compare median with IQR of not normally distributed data. The level of significance was set to 0.05. To quantify clinically significant improvements 24 months postoperatively the minimal clinically important difference (MCID) was calculated using the half-standard deviation method for VAS, IKDC and KOOS as previously described [15].

## Results

### Demographic patient data and complications

**Fig. 2:**
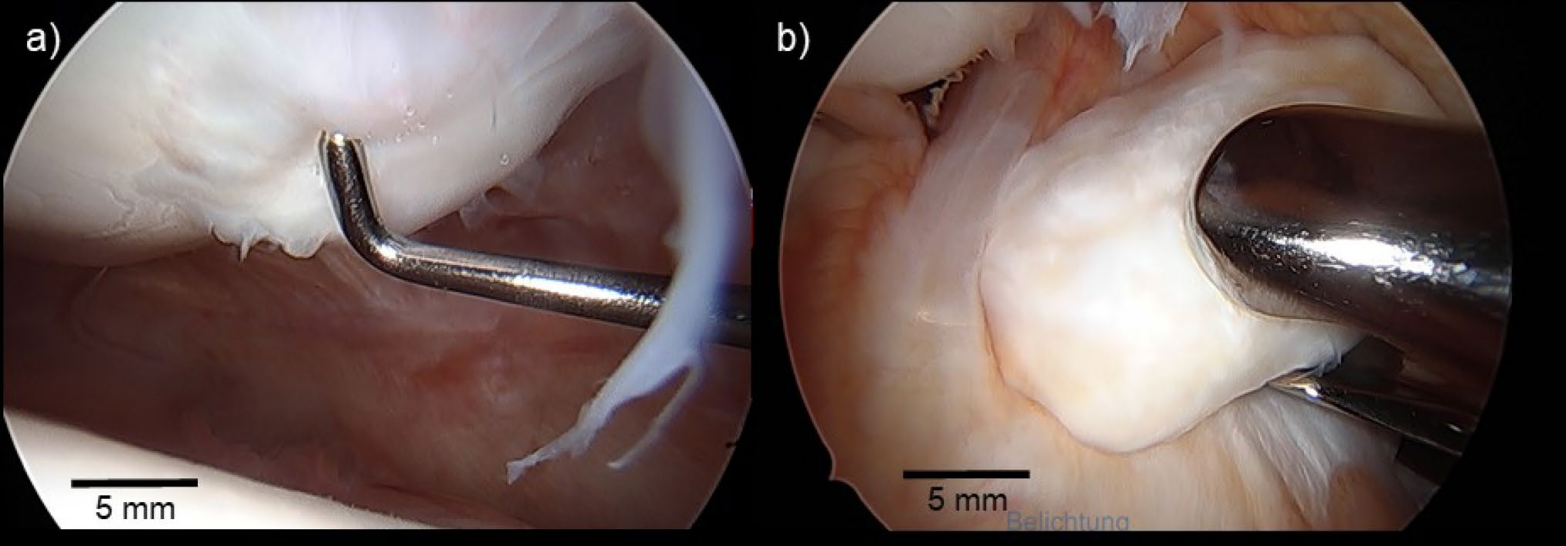
**a** Intraoperative view on former cartilage lesion with complete, albeit hypertrophic defect filling after 3 months. **b** Reoperation was indicated due to a loose body within the joint, that did not originate from the treated cartilage lesion (Case 11)

Between June 2020 and June 2021 11 patients (12 operations) were included in the study. The outcome of 11 operations (8 females/3 males) was followed up until 24 months postoperatively. One case was excluded due to a conversion to total knee arthroplasty 23 months after cartilage repair surgery. The mean patient age in the 11 included cases was 42.4 ± 11.7 years, average BMI was 30.9 ± 7.0 kg/m^2^ and mean lesion size was 3.2 ± 1.6 cm^2^. In 5 cases the defect was located on the medial femoral condyle, in 2 cases at the patellar, in 3 cases at the trochlear and in 1 case at the lateral femoral condyle. Of the 11 included cases 3 were treated all arthroscopic and 8 required a mini-open arthrotomy for the implantation of minced-cartilage. One patient received a revision knee arthroscopy 3 months after the initial autologous shaver-based minced cartilage implantation procedure at the patella due to a free loose body within the joint. Intraoperative investigation revealed that the loose body did not originate from the side of cartilage repair and early macroscopic result of MCI, showing complete, hypertrophic lesion filling could be documented in this instance (Fig. 2). Apart from this no reoperations or complications were observed following treatment of focal cartilage lesions of the knee with MCI.

### Patient reported outcome

**Fig. 3:**
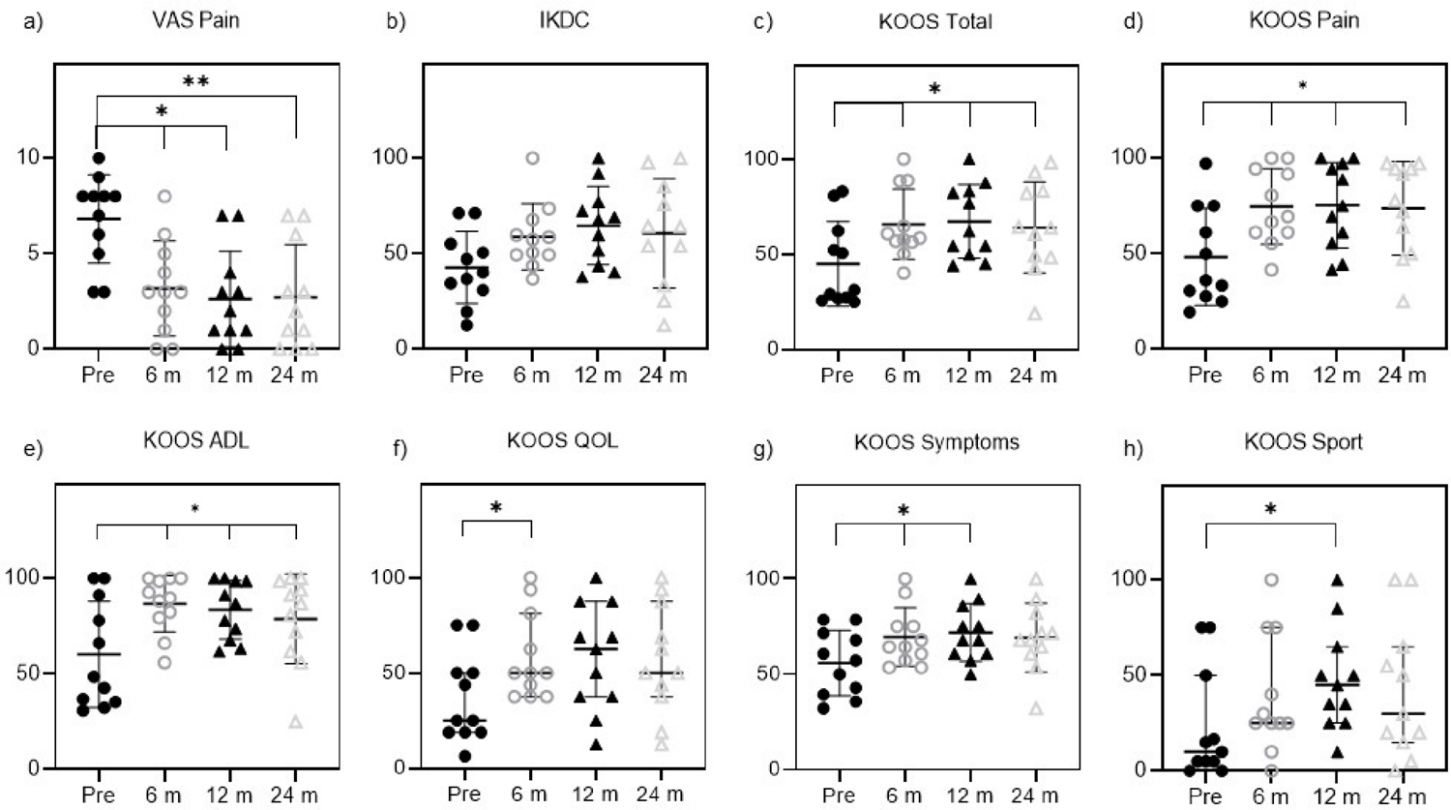
Overview of evaluated patient reported outcome measures Visual Analogue Scale Pain (VAS Pain), International Knee Documentation Committee Questionnaire (IKDC), Knee Injury and Osteoarthritis Outcome Score (KOOS) Total and KOOS subscores Pain, Activities of Daily living (ADL), Quality of Life (QOL), Symptoms and Sport. Comparison of preoperative values (Pre) to 6 months (6 m), 12 months (12 m) and 24 months (24 m) postoperatively (*p < 0.05, **p < 0.01)

The median pain under load, as reported by patients using the Visual Analog Scale (VAS), showed a statistically significant reduction from the preoperative assessment to all postoperative time points. Preoperatively, the median VAS score was 8.0 (IQR 5.0–8.0). At 6 months postoperatively, the score decreased to 3.0 (IQR 1.0–5.0, *p* = 0.0193), further declining to 2.0 at both 12 months (IQR 1.0–4.0, *p* = 0.0193) and 24 months (IQR 0.0–6.0, *p* = 0.0016) (Fig. 3a). The mean IKDC score improved but did not reach statistical significance (Fig. 3b). KOOS total and all KOOS subscores improved at all time points from pre- to postoperative. Statistically significant improvement was observed for KOOS total at 6 months (58.5 (56.4–88.4), *p* = 0.031), 12 months (62.2 (49.9–83.2), *p* < 0.031) and 24 months (63.9 (48.4–83.2), *p* = 0.04, Fig. 3c) postoperative, KOOS Pain from preoperative to 6 months (74.8 ± 19.8, *p* = 0.022), 12 months (75.3 ± 22.3, *p* = 0.012) and 24 months (73.7 ± 24.6, *p* = 0.028, Fig. 3d) postoperative, for KOOS ADL from preoperative to 6 months (86.6 ± 14.7, *p* = 0.019), 12 months (83.6 ± 15.3, *p* = 0.019) and 24 months (78.7 ± 23.5, *p* = 0.039, Fig. 3e) postoperative, for KOOS QOL from preoperative to 6 month (50.0 (37.5–81.3), *p* = 0.031) postoperative (Fig. 3f), for KOOS Symptoms from preoperative to 6 months- (69.5 ± 15.3, *p* = 0.036) and 12 months (71.8 ± 15.1, *p* = 0.045) postoperative (Fig. 3g) and KOOS Sport from preoperative to 12 months (45.0 (25.0–65.0), *p* = 0.05) postoperative (Fig. 3h). The MCID was reached for 81.8% (9/11) of patients considering VAS, 45.5% (5/11) considering IKDC, 63.6% (7/11) considering KOOS Total or KOOS Sport, 54.5% (6/11) considering KOOS QOL or KOOS Symptoms and 45.5% (5/11) considering KOOS ADL. For all KOOS subscores, as well as the IKDC a slight decrease from 12 to 24 months postoperatively was observed, that was not statistically significant.

### Clinical follow up examination

In 63.6% (7/11) of cases patients were able to passively flex the knee joint over 90° at 3 months follow up. This number increased to 90.1% (10/11) at 6 months follow up with all patients reaching more than 90° of flexion at 12 and 24 months follow up. At 12 months 63.6% (7/11) of patients had reached a degree of flexion that was similar to the contralateral knee, that proportion increased to 81.8% (9/11) at 24 months follow up. The mean Composite Score of the Y-Balance-Test increased from 83.7 ± 15.3 at 3-months-follow-up to 94.1 ± 14.6, 93.6 ± 10.6 and 99.8 ± 10.1 at 6-, 12- and 24-months-follow-up, respectively. However, this improvement did not reach statistical significance.

### MRI follow up investigation

MRI investigations performed 24 months after index surgery showed evidence of transplanted cartilage in the former cartilage lesion in 72.7% (8/11) of cases. The mean postoperative MOCART score was 51.4 ± 22.0. The T2 relaxation time of the regenerated cartilage was measured in 8 patients with definable regenerated cartilage. In these cases, the T2 relaxation time of regenerated cartilage in the cartilage lesion was compared to the surrounding non-damaged cartilage of the same patient. The resulting mean T2-index was calculated as 0.72 ± 0.18 (Table 1).

**Table 1:** Patient and lesion specific data as well as patient reported and radiological outcome in comparison of preoperative to 24 months postoperative

Case	Sex	BMI	Age	Lesion	Loc.	VAS		IKDC		KOOS Total		MOCART	T2 index
		kg/m^2^	years	cm^2^		Pre	Post	Pre	Post	Pre	Post		
1	f	24.5	33	1.5	RP	10	7	47.1	25.3	27.2	49.2	75	0.48
2	f	41.9	44	4.0	TRO	8	0	12.6	64.4	25.0	64.4	55	0.65
3	m	26.5	54	3.3	MFC	6	1	40.2	75.9	62.3	83.2	50	0.71
4	m	29.6	43	4.0	TRO	8	1	34.5	97.7	83.0	93.0	60	0.80
5	f	35.9	39	2.5	MFC	8	3	36.8	54.0	52.2	63.9	60	0.96
6	f	27.2	56	2.0	MFC	7	3	50.6	54.0	31.3	48.4	20	–
7	f	22.0	29	4.0	LFC	5	2	71.3	64.4	25.6	60.6	70	0.61
8	f	28.6	60	6.0	MFC	8	0	31.0	85.1	26.8	82.1	20	–
9	f	36.8	43	1.5	MFC	3	6	55.2	33.3	50.6	42.7	55	0.57
10	f	41.9	44	5.0	TRO	9	7	19.5	12.6	29.2	18.9	20	–
11	m	25.3	21	1.0	RP	3	0	71.3	100	80.7	98.2	80	0.96
M		30.9	42.4	3.2		8.0^a^	2.0^a^	42.7	60.6	31.3^a^	63.9^a^	51.4	0.72

*M* mean, *f* female, *m* male, *Lesion* lesion size, *Loc.* location of lesion, *RP* retropattelar, *TRO* trochlea, *MFC* medial femoral condyle, *LFC* lateral femoral condyle, *pre* preoperative, *post* postoperative

^a^Median values

## Discussion

The main findings of the study are, that the autologous shaver-based minced cartilage implantation for the treatment of focal cartilage lesions of the knee lead to a significant reduction of pain and improvement of function at 24 months follow up. In 3 of 11 patients no transplanted cartilage was observed during MRI examination 24 months after surgery. In patients with evidence of regenerated cartilage tissue the comparison of T2 relaxation times indicated a different structure of transplanted compared to intact hyaluronic cartilage of the same joints. However, in two cases qualitative MR morphological properties were similar to hyaline like cartilage, underlining the potential of the technique. Overall results were heterogenic with a number of excellent outcomes alongside observations of failed treatments.

Although MCI was first described in 1983 [1], high level randomized controlled studies on this surgical cartilage repair method and in particular on the autologous shaver-based minced cartilage implantation are absent. Therefore, while the autologous shaver-based minced cartilage technique is increasingly being adopted in clinical practice, no conclusions can be drawn regarding its effectiveness compared to established cartilage regeneration techniques. Two comparative studies on minced cartilage implantation (MCI) are available. The study by Cole et al. [16] demonstrated the superiority of MCI over MFX, however, it is based on an older generation of MCI techniques and may not reflect current advancements in the procedure. More recently, Behrendt et al. [17] reported superior outcomes for autologous matrix-induced chondrogenesis (AMIC) compared to MCI. This study warrants critical appraisal, as MCI was performed in conjunction with microfracture, but without the addition of orthobiologics. Previous studies emphasize the crucial role of orthobiologics in promoting chondrocyte function after outgrowth from minced cartilage in vitro [18]. Additionally, the retrospective nature of the study and the fact that MCI and AMIC patients were treated at different centres introduce potential confounding factors that could limit the validity of the comparison.

The studies by Massen et al. [19] and Runer et al. [20] provide encouraging 2- and 5-year results for minced cartilage implantation (MCI). The 5-year follow-up data were particularly anticipated, as they offer insight into the mid- to long-term efficacy of this technique. The findings confirm a sustained significant improvement in function and pain reduction compared to baseline. However, a decline in functional outcome was observed when compared to the 2-year follow-up results. Additionally, these studies included various MCI techniques, some of which incorporated collagen membranes, further complicating direct comparisons and generalizability of the results.

Of particular interest are three recently published case series that specifically investigate autologous shaver-based minced cartilage implantation [8–10]. These studies provide valuable insights into the clinical outcomes of this technique, offering a more targeted assessment. All three studies report encouraging results with a significant reduction of pain and functional improvement. When comparing our study with these case series, several key differences emerge. While our study had the smallest sample size, it is the only prospective investigation, adding methodological strength and reducing potential biases associated with retrospective designs. In terms of functional outcomes, the postoperative KOOS and IKDC scores in our study were lower compared to the other studies. Notably, Blanke et al. reported remarkably high postoperative IKDC scores, despite a larger mean defect size of 4.5 cm^2^ [8], whereas the defect size in our study averaged 3.2 cm^2^ and was larger than in the studies by Barbaret et al. and Schneider et al. [9, 10] Furthermore, it is important to consider that both Blanke et al. and Barbaret et al. reported their results at a mean follow-up of just over 1 year, whereas our study presents 2-year follow-up data, making direct comparisons challenging [8, 9]. The most striking differences between our cohort and those in the other studies are patient age and BMI, both of which were significantly higher in our study. These factors are well-known to influence cartilage repair outcomes and may partly explain the lower absolute postoperative scores in our cohort [21]. Additionally, our patients reported considerably lower baseline functional scores (IKDC and KOOS) and a markedly higher preoperative pain level. Despite the lower absolute postoperative scores, the observed improvement in functional outcomes in our study was comparable to that in the other studies. Thus, our findings in comparison to aforementioned studies demonstrate that older, overweight patients with poor baseline functional scores, will profit from autologous shaver-based minced cartilage implantation, since significant functional improvement and, most notably, pain reduction as observed. This finding is particularly relevant given that recent analyses from the German Cartilage Registry (Knorpelregister DGOU) have shown that cell-based cartilage regeneration techniques are often withheld from older patients in favour of simpler and less expensive, yet demonstrably less potent, procedures [22]. Thus, the observed improvement confirms that autologous shaver-based minced cartilage implantation represents a viable treatment option for this challenging patient group.

Follow up investigation with MRI after 24 months revealed that in 3 of 11 cases no regenerated cartilage was observed within the treated defect. A similar observation with 3 failed treatments in 29 cases according to postoperative MRI investigations was made following ACI [23]. The mean MOCART Score reported from this study (51.4 ± 22.0) was higher compared to an earlier study by Massen et al. (40.6 ± 21.1) [19], however the significantly longer follow up period in our study must be considered. While Schneider et al. and especially Barbaret et al. reported a higher postoperative MOCART Score following autologous shaver-based minced cartilage implantation [9, 10]. The smaller average defect size in the study by Barbaret et al. could be a contributing factor to the remarkably high MOCART score observed in their cohort. However, this assumption is contradicted by the findings of Blanke et al., who treated significantly larger defects but still reported a higher MOCART score than in our study. This suggests that defect size alone does not fully explain the differences. A more likely explanation lies in patient-specific factors such as higher age and BMI, which, as seen in the functional outcomes, may also influence cartilage regeneration and MRI morphology. Given the higher proportion of older and overweight patients, it is reasonable to assume that in our study a larger proportion of lesions were of degenerative origin, which could have negatively impacted the postoperative cartilage morphology. In this light, the T2-mapping results of our study must be analysed with caution. Importantly, this is the first study to report T2-mapping for MCI, providing valuable new insights into the structural composition of the regenerated cartilage. In a similar approach to the present study Welsch et al. reported T2-Index values of 0.89 ± 0.12 for regenerated cartilage following MFX and 0.99 ± 0.16 for regenerated cartilage following ACI [24]. Both reported T2-indeces were closer to 1.0 than in our study. Given the possible negative impact of age and BMI in our study, it is necessary to look beyond the mean values and analyse individual cases. Notably, in two cases of our study, a T2-Index of 0.96 was observed, which suggests that under certain conditions, the regeneration of hyaline-like cartilage following autologous shaver-based minced cartilage implantation may be possible.

This hypothesis is further supported by a number of preclinical studies. In this regard it was shown, that chondrocytes from fragmented cartilage tissue showed outgrowth into 3D-scaffolds and led to hyaline-like regenerated cartilage when implanted into full thickness cartilage defects in a goat model [25]. In an in vitro study superior cell proliferation and matrix production of minced cartilage tissue compared to isolated chondrocytes—the mainstay of ACI treatment - was shown [26]. 

While in the present study the majority of treated patients reached the MCID in functional outcome and especially postoperative pain reduction, a relatively high proportion of treatment failure was observed. In this regard a number of controversies on MCI still exist: The most suitable operative technique and especially the utilization of an arthroscopic shaver for harvesting and fragmentation of cartilage [27–29], the best side for cartilage harvest, the role of orthobiologics and probably most importantly, the selection of suitable patients since an enormous variety of cartilage quality depending on the minced-cartilage-donor has been observed in vitro [18]. 

### Limitations

A limitation of this study is the absence of a comparison to patients who received an established cartilage repair treatment. We minimized influencing factors by strict exclusion of patients who received concomitant surgeries like patella stabilization or realignment surgery, osteotomies and meniscal- or ligament surgeries of the knee. A second limitation is that in connection with the relatively small number of included patients, the heterogeneity of our study group concerning patient age, lesion size as well as location of cartilage defect, lead to a high dispersion of the results and could be one factor contributing to a relatively high proportion of treatment failure observed in this study. Furthermore, the study population was too heterogeneous to conclude on factors correlating with better or worse outcome. Finally, the follow up of 24 months is too short to definitely conclude on the efficacy of the described procedure, as according to the literature most cartilage repair techniques including MFX show positive effects at 24 months follow up and especially because a slight decrease of functional outcome following MCI from 12 to 24 months follow up was observed in this study.

## Conclusion

Treatment of focal cartilage lesions of the knee with the autologous shaver-based minced cartilage implantation provides adequate pain relief and improved functional outcome. Radiologic results indicated that in 3 out of 11 cases no transplanted cartilage tissue was found in the lesion. T2 mapping showed that, on average, the regenerated cartilage exhibited a structure different from hyaline cartilage. However, in two cases, the qualitative MR morphology of regenerated cartilage was similar to hyaline cartilage, indicating the potential of autologous shaver-based minced cartilage implantation. The heterogenic results have to be further analysed in order to improve patient selection and operative technique.

## Supplementary Information

Below is the link to the electronic supplementary material.

Supplementary Material 1

## Data Availability

The datasets generated during and analysed during the current study are available from the corresponding author on reasonable request.

## References

[1] Albrecht FH (1983) Closure of joint cartilage defects using cartilage fragments and fibrin glue. Fortschr Med 101:1650–1652 6605904

[2] Salzmann GM, Calek A-K, Preiss S (2017) Second-Generation autologous minced cartilage repair technique. Arthrosc Tech 6:e127–e131. 10.1016/j.eats.2016.09.011 28373950 10.1016/j.eats.2016.09.011 PMC5368339

[3] Armoiry X, Cummins E, Connock M et al (2019) Autologous chondrocyte implantation with chondrosphere for treating articular cartilage defects in the knee: an evidence review group perspective of a NICE single technology appraisal. PharmacoEconomics 37:879–886. 10.1007/s40273-018-0737-z 30426462 10.1007/s40273-018-0737-z

[4] Saris DBF, Vanlauwe J, Victor J et al (2008) Characterized chondrocyte implantation results in better structural repair when treating symptomatic cartilage defects of the knee in a randomized controlled trial versus microfracture. Am J Sports Med 36:235–246. 10.1177/0363546507311095 18202295 10.1177/0363546507311095

[5] Saris D, Price A, Widuchowski W et al (2014) Matrix-applied characterized autologous cultured chondrocytes versus microfracture: two-year follow-up of a prospective randomized trial. Am J Sports Med 42:1384–1394 24714783 10.1177/0363546514528093

[6] Brittberg M, Recker D, Ilgenfritz J et al (2018) Matrix-applied characterized autologous cultured chondrocytes versus microfracture: five-year follow-up of a prospective randomized trial. Am J Sports Med 46:1343–1351. 10.1177/0363546518756976 29565642 10.1177/0363546518756976

[7] Knutsen G, Drogset JO, Engebretsen L et al (2016) A randomized multicenter trial comparing autologous chondrocyte implantation with microfracture: long-term follow-up at 14 to 15 years. JBJS 98:1332–1339 10.2106/JBJS.15.01208 27535435

[8] Blanke F, Warth F, Oehler N et al (2024) Autologous platelet-rich plasma and fibrin-augmented minced cartilage implantation in Chondral lesions of the knee leads to good clinical and radiological outcomes after more than 12 months: a retrospective cohort study of 71 patients. J Exp Orthop 11:e70051. 10.1002/jeo2.70051 39415804 10.1002/jeo2.70051 PMC11480521

[9] Barbaret A, Wein F, Jacquet C et al (2025) One-stage minced cartilage autograft with platelet-rich plasma improves early clinical outcomes: a multicentric retrospective study. J Exp Orthop 12:e70162. 10.1002/jeo2.70162 39931147 10.1002/jeo2.70162 PMC11808269

[10] Schneider S, Ossendorff R, Walter SG et al (2024) Arthroscopic autologous minced cartilage implantation of cartilage defects in the knee: a 2-year follow-up of 62 patients. Orthop J Sports Med 12:23259671241297970. 10.1177/23259671241297970 39640183 10.1177/23259671241297970 PMC11618912

[11] Schneider S, Ossendorff R, Holz J et al (2021) Arthroscopic minced cartilage implantation (MCI): a technical note. Arthrosc Tech 10:e97–e101. 10.1016/j.eats.2020.09.015 33532215 10.1016/j.eats.2020.09.015 PMC7823081

[12] Irrgang JJ, Anderson AF, Boland AL et al (2001) Development and validation of the international knee documentation committee subjective knee form. Am J Sports Med 29:600–613. 10.1177/03635465010290051301 11573919 10.1177/03635465010290051301

[13] Roos EM, Roos HP, Lohmander LS et al (1998) Knee injury and osteoarthritis outcome score (KOOS)—development of a self-administered outcome measure. J Orthop Phys Ther 28:88–96. 10.2519/jospt.1998.28.2.88 10.2519/jospt.1998.28.2.88 9699158

[14] Marlovits S, Striessnig G, Resinger CT et al (2004) Definition of pertinent parameters for the evaluation of articular cartilage repair tissue with high-resolution magnetic resonance imaging. Eur J Radiol 52:310–319. 10.1016/j.ejrad.2004.03.014 15544911 10.1016/j.ejrad.2004.03.014

[15] Norman GR, Sloan JA, Wyrwich KW (2003) Interpretation of changes in health-related quality of life: the remarkable universality of half a standard deviation. Med Care 41:582–592. 10.1097/01.MLR.0000062554.74615.4C 12719681 10.1097/01.MLR.0000062554.74615.4C

[16] Cole BJ, Farr J, Winalski CS et al (2011) Outcomes after a single-stage procedure for cell-based cartilage repair: a prospective clinical safety trial with 2-year follow-up. Am J Sports Med 39:1170–1179 21460066 10.1177/0363546511399382

[17] Behrendt P, Eggeling L, Lindner A et al (2024) Autologous matrix-induced chondrogenesis provides better outcomes in comparison to autologous minced cartilage implantation in the repair of knee Chondral defects. Knee Surg Sports Traumatol Arthrosc. 10.1002/ksa.12387 39077845 10.1002/ksa.12387

[18] Gebhardt S, Zimmerer A, Balcarek P et al (2023) The influence of arthroscopic shaver mincing and Platelet-Rich plasma on chondrocytes of intraoperatively harvested human cartilage. Am J Sports Med 3635465231181633. 10.1177/03635465231181633 10.1177/03635465231181633 PMC10394959 37449659

[19] Massen FK, Inauen CR, Harder LP et al (2019) One-step autologous minced cartilage procedure for the treatment of knee joint Chondral and osteochondral lesions: a series of 27 patients with 2-year follow-up. Orthop J Sports Med 7:2325967119853773 31223628 10.1177/2325967119853773 PMC6566484

[20] Runer A, Ossendorff R, Öttl F et al (2023) Autologous minced cartilage repair for Chondral and osteochondral lesions of the knee joint demonstrates good postoperative outcomes and low reoperation rates at minimum five-year follow-up. Knee Surg Sports Traumatol Arthrosc 31:4977–4987. 10.1007/s00167-023-07546-1 37634136 10.1007/s00167-023-07546-1 PMC10598129

[21] Robb CA, El-Sayed C, Matharu GS et al (2012) Survival of autologous osteochondral grafts in the knee and factors influencing outcome. Acta Orthop Belg 78:643–651 23162961

[22] Gebhardt S, Vollmer M, Zimmerer A et al (2024) Factors affecting choice of surgical treatment of cartilage lesions of the knee: an analysis of data from 5143 patients from the German cartilage registry (KnorpelRegister DGOU). Orthop J Sports Med 12:23259671241255672. 10.1177/23259671241255672 39070901 10.1177/23259671241255672 PMC11273558

[23] Siebold R, Suezer F, Schmitt B et al (2018) Good clinical and MRI outcome after arthroscopic autologous chondrocyte implantation for cartilage repair in the knee. Knee Surg Sports Traumatol Arthrosc 26:831–839. 10.1007/s00167-017-4491-0 28258330 10.1007/s00167-017-4491-0

[24] Welsch GH, Trattnig S, Domayer S et al (2009) Multimodal approach in the use of clinical scoring, morphological MRI and biochemical T2-mapping and diffusion-weighted imaging in their ability to assess differences between cartilage repair tissue after microfracture therapy and matrix-associated autologous chondrocyte transplantation: a pilot study. Osteoarthritis Cartilage 17:1219–1227. 10.1016/j.joca.2009.03.018 19409295 10.1016/j.joca.2009.03.018

[25] Lu Y, Dhanaraj S, Wang Z et al (2006) Minced cartilage without cell culture serves as an effective intraoperative cell source for cartilage repair. J Orthop Res 24:1261–1270 16652342 10.1002/jor.20135

[26] Tsuyuguchi Y, Nakasa T, Ishikawa M et al (2018) The benefit of minced cartilage over isolated chondrocytes in atelocollagen gel on chondrocyte proliferation and migration. Cartilage 1947603518805205 10.1177/1947603518805205 PMC7755964 30311776

[27] Frings J, Baranowsky A, Korthaus A et al (2024) Arthroscopic Shaver-based harvest of minced cartilage results in reduced chondrocyte viability and reduced quality of cartilaginous repair tissue compared with open harvest and conventional fragmentation. Arthroscopy. 10.1016/j.arthro.2024.05.020 10.1016/j.arthro.2024.05.020 39230539

[28] Frings J, Baranowsky A, Keller J et al (2024) Author Reply to Regarding ‘Arthroscopic Minced Repair of Knee Cartilage Is Safely and Effectively Performed Using Arthroscopic Techniques’. Arthroscopy. 10.1016/j.arthro.2024.10.027. 10.1016/j.arthro.2024.10.028 39505155

[29] Gebhardt S, Zimmerer A, Balcarek P et al (2024) Arthroscopic Shaver-based harvest of minced cartilage results in reduced chondrocyte viability and reduced quality of cartilaginous repair tissue compared with open harvest and conventional fragmentation. Arthroscopy. 10.1016/j.arthro.2024.10.026. 10.1016/j.arthro.2024.10.026 39491690

