# Supplementary figures and images for: Clinical and radiological 2-year results after autologous shaver-based minced cartilage implantation for cartilage lesions of the knee
Source: Arch Orthop Trauma Surg. 2025 Oct 10;145(1):465. doi: 10.1007/s00402-025-06010-8 (PMC12513964; doi:10.1007/s00402-025-06010-8)

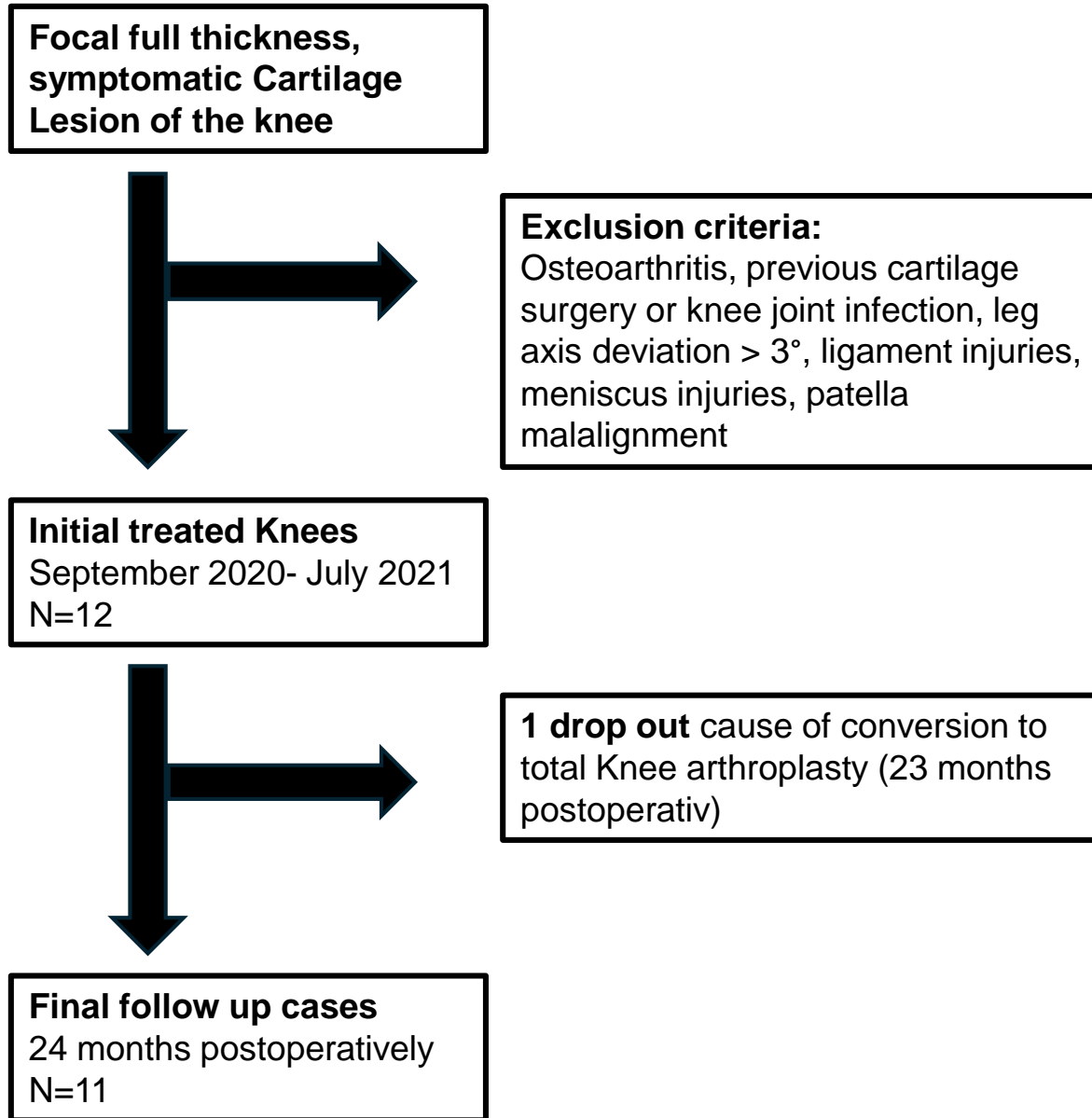

Flowchart: We did not hold record of patients who were not selected for the study

Supplement: Supplementary file 1 — Supplementary Material 1 [file 402_2025_6010_MOESM1_ESM.pdf]
